# Temperature and precipitation explain variation in metabolic rate but not frequency of gas exchange in Fijian bees

**DOI:** 10.1242/jeb.249948

**Published:** 2025-05-23

**Authors:** Carmen R. B da Silva, Julian E. Beaman, Marika Tuiwawa, Mark I. Stevens, Michael P. Schwarz, Rosalyn Gloag, Vanessa Kellermann, Lesley A. Alton

**Affiliations:** ^1^ School of Natural Sciences, Macquarie University, North Ryde, NSW 2109, Australia; ^2^ School of Biological Sciences, Monash University, Clayton, VIC 3800, Australia; ^3^ College of Science and Engineering, Flinders University, Bedford Park, SA 5042, Australia; ^4^ The University of the South Pacific, Herbarium, Viti Levu, Fiji; ^5^ Earth & Biological Sciences, South Australian museum, Adelaide, SA 5000, Australia; ^6^ School of Biological Sciences, University of Adelaide, SA 5005, Australia; ^7^ School of Life and Environmental Sciences, University of Sydney, Sydney, NSW 2050, Australia; ^8^ School of Agriculture, Biomedicine and Environment, La Trobe University, Melbourne, VIC 3000, Australia; ^9^ Centre for Geometric Biology, Monash University, Clayton, VIC 3800, Australia

**Keywords:** Metabolic cold adaptation, Krogh's rule, Discontinuous gas exchange, Hygric hypothesis, Thermal performance, Respiration, Tropical insects, Evolutionary physiology, Functional traits

## Abstract

Temperature and water availability are hypothesised to be important drivers of the evolution of metabolic rate and gas exchange patterns, respectively. Specifically, the metabolic cold adaptation (MCA) hypothesis predicts that cold environments select for faster temperature-specific metabolic rates to counter the thermodynamics of biochemical reactions, while the hygric hypothesis predicts that dry environments select for discontinuous gas exchange to reduce water loss. Although these two hypotheses consider different physiological traits and how they vary along different abiotic gradients, metabolic rate drives the frequency of gas exchange in insects meaning these two traits are inherently linked. Despite this link, the MCA and hygric hypotheses are rarely considered together and the extent to which metabolic rates and frequency of gas exchange vary and co-vary across climatic gradients remains unclear. We tested the MCA and hygric hypotheses within a species of endemic Fijian bee, *Homalictus fijiensis*, and among four Fijian bee species across an altitudinal gradient of 1100 m (highlands are colder and wetter than lowlands). We found an MCA-like pattern within *H. fijiensis* and among Fijian bee species, where bees from colder environments had higher metabolic rates than bees from warmer environments when measured at 25°C, but precipitation also explained variation in metabolic rate. However, we did not find support for the hygric hypothesis within *H. fijiensis* or among species (frequency of gas exchange was not negatively correlated with precipitation). The relationship between metabolic rate and frequency of gas exchange was steeper for species that occupied lower elevations on average, suggesting it is possible that these two traits can evolve independently of each other despite being positively correlated.

## Teaser

**Summary:** Temperature and precipitation explain variation in Fijian bee metabolic rate, partially supporting the metabolic cold adaptation hypothesis. Aridity does not explain variation in frequency of gas exchange, rejecting the hygric hypothesis.

## INTRODUCTION

Variation in climate shapes species' physiological traits and geographic distributions (Diamond and Martin, 2021; Hoffmann and Sgro, 2011; Kellermann et al., 2012). For terrestrial ectotherms in particular, environmental temperature and aridity are hypothesised to be important abiotic drivers of the evolution of key physiological traits such as metabolic rate and respiratory patterns (Chown et al., 2011; White et al., 2007). These two traits underpin the adaptive hypotheses known as the metabolic cold adaptation (MCA) and hygric hypotheses, which aim to explain how and why metabolic rate and respiratory patterns, respectively, vary among populations and species that occupy different climatic niches (Addo-Bediako et al., 2002; Alton et al., 2017; Chown et al., 2006; Terblanche et al., 2010; White et al., 2007, 2012). These two hypotheses have been hotly debated (Addo-Bediako et al., 2002; Marais et al., 2005; White et al., 2007; Chown et al., 2011; Willot et al., 2023), and studies that seek to understand the environmental drivers of variation in metabolic rate and respiratory patterns rarely consider the MCA and hygric hypotheses together, even though they are inherently linked (but see Davis et al., 2000; Terblanche et al., 2010).

Many insects breathe discontinuously, opening and closing their spiracles to alternate between phases of breath-holding and gas exchange with the atmosphere, and the frequency of these discontinuous gas exchange cycles increases with metabolic rate (Contreras and Bradley, 2009, 2010; Schimpf et al., 2012; Gibbs and Johnson, 2004; White et al., 2007). A similar relationship between gas exchange frequency and metabolic rate is observed for species that breathe cyclically without closing their spiracles (Marais et al., 2005). However, whether discontinuous gas exchange patterns are adaptive remains a topic of debate (Gefen and Matthews, 2021; Gibbs and Johnson, 2004; Matthews and White, 2011; Oladipupo et al., 2022; Rowe et al., 2022; White et al., 2007).

Given the physiological link between metabolic rate and frequency of discontinuous gas exchange, it is possible that selection on either trait would result in a correlated change in the other, or a change in the relationship between them. Thus, to better understand the evolution of one trait requires consideration of the other to determine how environments have shaped trait variation and covariation. The metabolic cold adaptation hypothesis (Wohlschlag, 1960), or Krogh's rule (Gaston et al., 2009), predicts that organisms from cold environments (such as high latitudes and altitudes) will have higher metabolic rates than those from warm environments, when measured at the same temperature. This pattern is expected to arise as a mechanism to maintain physiological function in cold environments because acute exposure to colder temperatures causes biochemical reaction rates to slow (Addo-Bediako et al., 2002; Gaston et al., 2009; Krogh, 1916; White et al., 2012). However, in terrestrial organisms, the pattern described by the MCA hypothesis may also emerge as a consequence of organisms depressing their metabolic rate in response to high temperatures to prevent desiccation (Addo-Bediako et al., 2001). This is because high air temperatures result in high vapour pressure deficits (VPDs) that can lead to higher rates of water loss from wet respiratory surfaces (White et al., 2007). Organisms living in warmer environments that are also more desiccating could therefore slow their metabolic rate, reduce their gas exchange frequency and breathe discontinuously. The hygric hypothesis makes the explicit prediction that discontinuous gas exchange in resting tracheate arthropods, such as insects, is an adaptation to limit respiratory water loss. As such, drier environments are expected to favour arthropods that breathe discontinuously and, by extension, those that hold their breath for longer (i.e. have a lower frequency of gas exchange) (Buck et al., 1953; Chown et al., 2011; Terblanche et al., 2010; White et al., 2007).

In insects, both the MCA and hygric hypotheses tend to be supported by broad-scale comparative analyses (Addo-Bediako et al., 2002; White et al., 2007), but not always (Messamah et al., 2017). Laboratory natural selection experiments that manipulate temperature have also failed to replicate the pattern described by the MCA hypothesis in *Drosophila* (Alton et al., 2017, 2024; Mallard et al., 2018). Such research indicates that temperature alone may not be responsible for driving the evolution of metabolic rate. The hygric hypothesis similarly garners equivocal support (Chown, 2002), and alternative adaptive hypotheses propose that discontinuous gas exchange has evolved as a mechanism to improve gas exchange in underground environments, or to reduce oxidative tissue damage (Buck et al., 1953; Chown et al., 2006; Hetz and Bradley, 2005; Lighton, 1998). It has also been suggested that discontinuous gas exchange is not an adaptation to environmental conditions, but instead arises as a consequence of neural control of gas exchange during rest (Matthews and White, 2011; Rowe et al., 2022). Given that there is mixed support for both the MCA and hygric hypotheses, and that selection rarely acts on single traits independently (Blows, 2007; Calsbeek and Irschick, 2007; Lande and Arnold, 1983), we propose that these hypotheses should be considered simultaneously to better understand how metabolic rate and respiratory patterns vary and co-vary along climatic gradients.

**Fig. 1.**
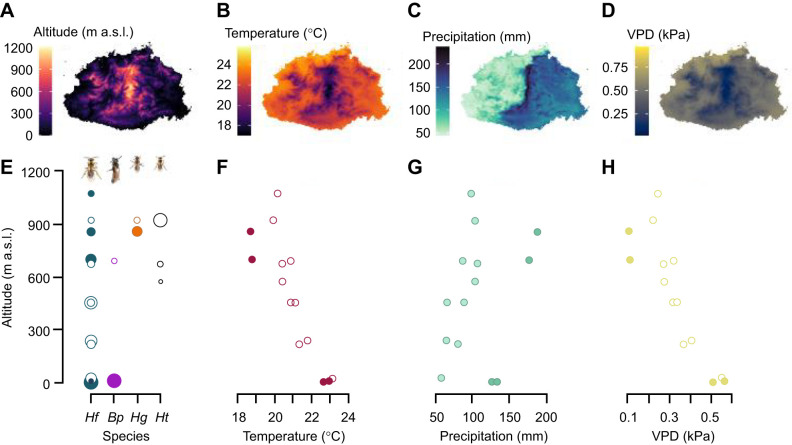
**Abiotic variables across Viti Levu, Fiji.** (A) Altitude (a.s.l., above sea level), (B) mean temperature of the coolest and driest month (July), (C) precipitation of the driest month (July) and (D) vapour pressure deficit (VPD) of the driest month (July). (E) Collection altitude of each species (Hf, *Homalictus fijiensis*; Bp, *Braunsapis puangensis*; Hg, *Homalictus groomi*; Ht, *Homalictus tuiwawae*) where circle size indicates the magnitude of the sample size. (F) Relationship between altitude and environmental temperature. (G) Relationship between altitude and precipitation. (H) Relationship between altitude and VPD. Filled circles indicate collection sites on the wet side of Viti Levu and open circles indicate sample sites on the dry side of Viti Levu.

Here, we examined how metabolic rate and frequency of discontinuous gas exchange vary and co-vary within and among bee species that breathe discontinuously (da Silva et al., 2021) and exist along an altitudinal gradient on the tropical mountainous island of Viti Levu, Fiji. Altitudinal gradients are useful systems in which to test hypotheses concerning climatic drivers of trait variation because the spatial rate of change in climate is greater along altitudinal gradients than latitudinal gradients, while habitat composition and photoperiod vary less (Jump et al., 2009; Halbritter et al., 2018). Viti Levu is divided by a mountain range that runs north to south and the highest peak is 1328 m above sea level (a.s.l.). The highlands in the centre of the island are characterised by cloud forests that are ∼5°C cooler and receive ∼100 mm more precipitation per month than the coastal lowlands (Fig. 1). Because of the mountain range that divides Viti Levu, there is also a rain shadow on the western side of the island that receives significantly less rainfall than the eastern side (Fig. 1C). The bee species that inhabit Viti Levu occupy different altitudinal ranges: the endemic *Homalictus fijiensis* is found along the entire altitudinal gradient while 22 other endemic *Homalictus* species are restricted to the highlands, and the invasive *Braunsapis puangensis*, is generally restricted to the lowlands (da Silva et al., 2016a,b; Dorey et al., 2020; Groom et al., 2013).

**Fig. 2.**
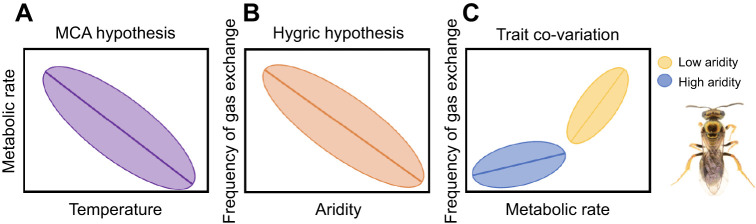
**Conceptual illustration of the relationship between traits and climate expected as per the metabolic cold adaptation hypothesis, hygric hypothesis and how traits might co-vary across an aridity gradient.** (A) Relationship between metabolic rate and temperature if the metabolic cold adaptation (MCA) hypothesis is supported. (B) Relationship between frequency of gas exchange and aridity (inverse of precipitation or vapour pressure deficit, VPD) if the hygric hypothesis is supported. (C) Change in relationship between metabolic rate and frequency of gas exchange depending on aridity (precipitation or VPD). Where there is reduced selective pressure to avoid desiccation, we expect that selective pressure on metabolic rate and frequency of gas exchange will be relaxed, resulting in a steep relationship between metabolic rate and frequency of gas exchange in areas of high precipitation or low VPD. *Homalictus fijiensis* photo credit: James B. Dorey.

To examine the climatic drivers of variation and co-variation in metabolic rate and respiratory patterns of bees, we collected individuals of *H. fijiensis*, *Homalictus groomi*, *Homalictus tuiwawae* and *B. puangensis* from multiple sites on either side of the rain shadow along the altitudinal gradient of Viti Levu (Fig. 1E). We measured the metabolic rates of 208 individual bees at 25°C (mean environmental temperature during September collection period) as rates of carbon dioxide production using flow-through respirometry. We examined the relationships between metabolic rate and frequency of gas exchange of bees and the climatic variables at their collection site using a strong inference framework whereby a set of models was selected *a priori* based on the explicit predictions of the MCA and hygric hypotheses (see Table 1 and Box 1 for a list of models tested). We accepted support for the MCA hypothesis if bees from cooler climates had higher metabolic rates and the only climate variable in the best model was temperature (Fig. 2A). We accepted support for the hygric hypothesis if bees from drier climates had a lower frequency of gas exchange and the only climate variable in the best model was either VPD or precipitation (Fig. 2B), both of which are assumed to be indicative of aridity. In addition to testing the predictions of the MCA and hygric hypotheses, we also tested the hypothesis that frequency of gas exchange and metabolic rate are positively correlated, but that the strength of the relationship between these two traits varies depending on the aridity of the environment (Fig. 2C). All else being equal, insects with higher metabolic rates breathe more frequently (Contreras and Bradley, 2009, 2010; Schimpf et al., 2012), but we expect that in drier environments there will be stronger selection for insects with high metabolic rates to maintain lower frequencies of gas exchange to minimise water loss, which will result in a shallower relationship between frequency of gas exchange and metabolic rate. In wetter environments where the risk of desiccation is lower, we expect that selection on low frequencies of gas exchange will be relaxed, resulting in a steeper relationship between frequency of gas exchange and metabolic rate.

**Box 1. Expansion of hypothesis rationale for model curation**

**Hypotheses to explain variation in metabolic rate (log_10_ transformed to satisfy linear mixed model requirements):**

1. Individuals from colder environments will have higher metabolic rates than individuals from warmer environments. MCA hypothesis supported.

2. Individuals from low precipitation environments have the lowest metabolic rates to avoid respiratory water loss. MCA hypothesis rejected.

3. Drying power of the air (vapour pressure deficit, VPD) best explains variation in metabolic rate, where individuals from high VPD regions (warm and dry) have the lowest metabolic rates to decrease respiratory water loss. MCA hypothesis rejected.

4. Because of the ‘wet’ and ‘dry’ side of Viti Levu (Fig. 1C), temperature and precipitation independently impact metabolic rate, where both low temperatures and high precipitation select for low metabolic rates. However, thermal environments can vary in their level of precipitation depending on location (wet or dry side and altitude). MCA-like pattern is observed, but precipitation is also an important predictor of metabolic rate.

5. Null hypothesis: metabolic rate changes across altitude, but some other abiotic variable is shaping variation in metabolic rate. MCA hypothesis rejected.

**Hypotheses to explain variation in frequency of gas exchange:**

6. Individuals from low precipitation environments will have the lowest frequencies of gas exchange to reduce water loss. Hygric hypothesis supported.

7. Environmental temperature impacts frequency of gas exchange as a result of the direct thermodynamic effects of temperature on performance (Angilletta et al., 2010), where rates of gas exchange increase with warming temperatures. Hygric hypothesis rejected.

8. Drying power of the air (VPD) best explains variation in frequency of gas exchange, where individuals from high VPD environments have the lowest frequency of gas exchange to decrease respiratory water loss. Hygric hypothesis supported.

9. Temperature and precipitation independently shape variation in frequency of gas exchange, where both high temperatures and low precipitation select for low frequencies of gas exchange, but locations can vary in their temperature/precipitation combination. Hygric hypothesis supported, but temperature also plays an important role in explaining variation in frequency of gas exchange.

10. Null hypothesis: frequency of gas exchange changes across altitude, but some other abiotic variable (other than temperature, precipitation and aridity) is shaping variation in frequency of gas exchange.

**Hypotheses to explain co-variation between metabolic rate and frequency of gas exchange:**

11. The relationship between metabolic rate and frequency of gas exchange will be impacted by the environmental temperature species inhabit, where the frequency of gas exchange–metabolic rate relationship will be steeper in cooler environments than warmer environments as the selective pressure to increase metabolic rate in cold environments will be greater than the selective pressure to decrease frequency of gas exchange.

12. The relationship between metabolic rate and frequency of gas exchange will be impacted by aridity (VPD), where the frequency of gas exchange–metabolic rate relationship will be steeper in low VPD environments as selective pressure on frequency of gas exchange will be relaxed in low VPD environments.

13. The relationship between metabolic rate and frequency of gas exchange will be impacted by precipitation, where the selective pressure to reduce frequency of gas exchange will be greater in dry environments than the selective pressure to reduce metabolic rate.

14. The relationship between metabolic rate and frequency of gas exchange is best explained by the additive effects of temperature and precipitation, where both warm temperatures and low precipitation independently select for low metabolic rates and frequency of gas exchange.

15. Null hypothesis: an interaction between metabolic rate and altitude better explains variation in frequency of gas exchange than the other models, suggesting that another variable better explains the covariation between metabolic rate and frequency of gas exchange.

**Table 1.** Hypotheses that were compared to test the metabolic cold adaptation hypothesis and the hygric hypothesis, and examine whether metabolic rate and frequency of gas exchange covary depending on the climates that species inhabit

Hypothesis predictor variable	MR (testing MCA hypothesis)	FGE (testing hygric hypothesis)	Trait covariance (does trait covariation depend on aridity?)
*T*_min_	–	○	○
*P*_dry_	○	+	FGE∼MR relationship is steepest in low *P*_dry_ environments
VPD	○	–	FGE∼MR relationship is steepest in low VPD locations
*T*_min_+*P*_dry_	If *T*_min_ has a negative effect on MR, an MCA-like pattern arises	If *P*_dry_ has a positive effect on FGE, a hygric-like pattern arises	NA
Altitude (null hypothesis)	○	○	○

*T*_min_, mean temperature of the coldest month (July; °C); *P*_dry_, precipitation of the driest month (July; mm); VPD, vapour pressure deficit of the driest month (July; kPa); MR, metabolic rate; MCA, metabolic cold adaptation; FGE, frequency of gas exchange. A negative symbol (−) indicates that the hypothesis predicts a negative relationship. A positive symbol (+) indicates that the hypothesis predicts a positive relationship. A circle (○) indicates that the hypothesis predicts a lack of a relationship between the response variable and the predictor variable.

## MATERIALS AND METHODS

### Animal collection

Bees were collected over 10 days in September 2019 on Viti Levu, Fiji. September falls within the dry season in Fiji, where precipitation and environmental temperatures are moderate (between the coolest and driest month, July, and the wettest and hottest month, February). Viti Levu is divided by a mountain range that runs north to south, which creates a rain shadow on the western side of the island that receives significantly less rainfall than the eastern side (Fig. 1C). We collected bees by sweep-netting from 16 sites along an altitudinal gradient of 6–1072 m a.s.l., with 10 of these sites being on the dry western side of the island and the other six sites being on the wet eastern side. Sites were sampled in a haphazard order over the 10 days and bees were collected by sweep-netting vegetation along road verges or in native vegetation. Individuals of *B. puangensis* were also collected directly from stem nests (*Homalictus* species inhabit ground nests which we did not collect from as this is extremely laborious and yields low sample sizes). The males and females of all species collected are known to be active year round and are multivoltine (Groom et al., 2013). Immediately upon capture, bees were placed in 50 ml plastic vials (10 cm in height and 2.5 cm in width) with foam lids and stored in an insulated container. Inside the vials, bees were provided with a small piece of paper towel dipped in a 20% sucrose solution. On the day of collection, bees were transported to the laboratory for their metabolic rate to be measured that same evening.

### Species summary

We collected four species: *Homalictus fijiensis* Perkins & Cheesman 1928, *Homalictus groomi* Dorey et al. 2019, *Homalictus tuiwawae* Dorey et al. 2019 and *Braunsapis puangensis* Cockerell 1928, across the elevational gradient. All of these species are relatively small in body size, but *B. puangensis* is slightly larger than the *Homalictus* species on average (Table S1). *Braunsapis puangensis* in Fiji have a communal nesting strategy (probably an evolutionary reversal in social evolution from eusociality to communal behaviour; da Silva et al., 2016a,b), and *Homalictus* species are also generally known to be communal (Houston, 2018). *Homalictus* species live in deep ground nests (often in sloped clay soils) and *B. puangensis* nests in pithy plant stems, frequently in managed gardens (da Silva et al., 2021). The *Homalictus* species in this study are endemic to Fiji; however, *B. puangensis* is an introduced species with a much broader latitudinal range (da Silva et al., 2021).

### Climatic variables

For each collection site, we downloaded historical climate data (1970–2000) at a 30 arcsec resolution from WorldClim2 (Fick and Hijmans, 2017) using the R package *raster* (https://CRAN.R-project.org/package=raster). As the MCA and hygric hypotheses make explicit predictions about how cool and dry climates shape metabolic rates and frequency of gas exchange, respectively, we characterised the climates at each collection site using the variables of mean temperature (°C) of the coolest month (*T*_min_; July), mean precipitation (mm) of the driest month (*P*_dry_; July), and mean vapour pressure (VP, kPa) of the driest month (July) (Fig. 1; Table S1). VP was used to calculate the vapour pressure deficit (VPD, kPa) (drying power of the air) for bees during the driest month (July) assuming that their respiratory surfaces are saturated with water vapour (saturated water vapour pressure, SVP, kPa) and that their body temperature matches that of the environment (*T*_min_, mean temperature of the coolest month, °C) using Eqn 1 (Monteith and Unsworth, 2013) and Eqn 2:
(1)

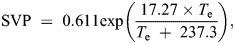

(2)

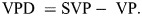


We tested the accuracy of the WorldClim2 environmental temperature data, which is based on a combination of weather station data, satellite data and elevational maps, by placing HOBO temperature data loggers (www.hobodataloggers.com.au) across the altitudinal gradient at 10, 200, 700, 900 and 1072 m for a period of 2–3 weeks in September–October 2019 (Fig. S1). The highland was about 5°C cooler and received an additional 100 mm precipitation each month compared with the lowland region, according to the WorldClim2 data. Based on the HOBO loggers, the mean temperature at the lowland site (Suva, 10 m a.s.l.) during the measurement period was 25.4°C and the mean temperature of the highland site (Tel tower near Nadarivatu, 1072 m) was 20.05°C. As the HOBO temperature data and WorldClim2 data are well aligned, and because we were unable to measure temperature at each collection site, we used WorldClim2 data to collect environmental data from all sites.

### Trait measurements

We measured the metabolic rate and frequency of gas exchange of individual bees by measuring their rates of carbon dioxide production (*V̇*_O_2__, ml min^–1^) at 25°C (mean environmental temperature across sites during collection in September) using a seven-channel flow-through respirometry system. The respirometry system was supplied with room air that was pushed (using an air pump) through columns of soda lime and Drierite^®^ to remove carbon dioxide and water vapour, respectively. Flow rate through each of the seven channels was regulated to 100 ml min^–1^ by a mass flow controller (Aalborg, Model GFC17, Orangeburg, NY, USA). The volumetric flow rate produced by the flow controllers was measured using a Gilian Gilibrator-2 NIOSH Primary Standard Air Flow Calibrator with a low-flow cell (Sensidyne, LP, St Petersburg, FL, USA) and corrected to standard temperature and pressure, STP (101.3 kPa and 0°C). To prevent bees from desiccating during measurements, the air was re-humidified after the flow controllers by passing the air through a humidifying chamber (a syringe of wetted cotton). The humidified air then flowed through a respirometry chamber (2.5 ml plastic syringe) containing an individual bee. Respirometry chambers were situated inside a temperature-controlled cabinet that maintained air temperature to 25±1°C and kept bees in the dark (bees are attracted to light so they were kept in the dark to minimise activity). The excurrent air from the respirometry chamber then flowed through one of seven infrared CO_2_/H_2_O gas analysers (LI-COR, Model LI-840A, Lincoln, NE, USA) that were calibrated with precision span gases (5.0 and 30.4 ppm CO_2_, Alphagaz – Air Liquide, Melbourne, VIC, Australia) and recorded CO_2_ concentrations at a sampling rate of 1 Hz using the PowerLab (ADInstruments) data acquisition system.

The fractional CO_2_ concentration of the excurrent air (*F*e_CO_2__) from each respirometry chamber was recorded for 50 min. The fractional CO_2_ concentration of the incurrent air when it was stable was measured for 2 min before and after each 50 min measurement period with an empty chamber. We corrected for analyser drift by fitting a linear model to these data to estimate the CO_2_ concentration of the incurrent air during the 50 min measurement period (*F*i_CO_2__). The rate of carbon dioxide production (*V̇*_CO_2__, ml min^–1^) was then calculated using Eqn 3 (Lighton, 2018) where FR is the flow rate (ml min^–1^) corrected to STP (101.3 kPa and 0°C) accounting for water vapour dilution:
(3)

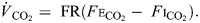
The first and last 10 min of the 60 min measurement period were considered to be a settling in/out period and were discarded from analysis (however, almost all bees breathed discontinuously within a few seconds of being placed in the chamber). We measured metabolic rate over complete gas exchange cycles (from the start of the first breath until the end of the seventh breath; average measurement time: 13.6 min). Some individuals exchanged gas so slowly that seven was the maximum number of respiratory cycles that we could measure within the 40 min measurement period. Thus, we measured metabolic rate over seven respiratory cycles for each individual (see Fig. S2). Seven respiratory cycles is adequate for estimating metabolic rate without a large measurement error (Winwood-Smith and White, 2018).

Immediately following metabolic rate measurements, bees were preserved in 100% ethanol to be sexed and weighed at a later date. Bees were sexed by visual assessment by examining the number of antennal segments (males have 13 and females have 12). Prior to weighing, the right hindleg of each individual was detached for species identification via CO1 barcoding (see below). Dry mass (rather than wet mass because of preservation in ethanol) was measured 2 months after metabolic rate measurements with specimens dried at 60°C for 48 h prior to being weighed (XP2U Ultra Micro Balance, Mettler Toledo, Greifensee, Switzerland).

Bees were measured during the evening on the day that they were collected from the field. Because of the logistical challenges of bee collection on Viti Levu, bees could not be collected from all sites each day. Consequently, on some nights, bees from multiple sites could be measured, but on other nights only bees from one site could be measured. As the species and sex of the bees measured each night were mostly unknown (see below), measurements were conducted blind to the species and sex of bees in most cases (*B. puangensis* is easily differentiated from *Homalictus* species). After species were identified (see below), only those species with sample sizes greater than 10 were retained for analysis. In total, we measured 208 individuals of four species: *H. fijiensis n*=125, *H. tuiwawae n*=23, *H. groomi n*=16, and *B. puangensis n*=44 (see Table S1 for the climate that each species was collected from and their mean body mass).

### Species identification

*Homalictus* species living at high altitudes are cryptic and, in many cases, can only be identified via male genitalia morphology, or via genetic comparison. To identify our *Homalictus* species collected from altitudes above 300 m a.s.l., we sent tissue samples (the right hindleg) to the Canadian Centre for DNA Barcoding (CCDB) at the University of Guelph, ON, Canada, to have the mtDNA COI gene fragment sequenced. A total of 84 samples were DNA barcoded using single-molecule real-time sequencing (SMRT49) using the PacBio Sequel platform (Pacific Biosciences, Menlo Park, CA, USA). Sequences were aligned to existing COI sequences taken from species identified by Dorey et al. (2020, 2021). The invasive bee species *B. puangensis*, which is generally restricted to the lowlands, is similar in size to *H. fijiensis*, but is distinguishable from *Homalictus* species by its black cuticle and white facial marking (da Silva et al., 2016a,b, 2021). Specimens below 300 m a.s.l. were not sequenced because they can be identified via morphology: 98% of *Homalictus* bees in this elevational band are *H. fijiensis*, which have a green thorax (Dorey et al., 2020). The extremely rare collection of *H. hadrander* (blue thorax; 1%) and *H. groomi* (pink thorax; 0.5%) can easily be distinguished from *H. fijiensis*. *Homalictus* species I (yet to be formally identified; green thorax; 0.5%) is the only species that might have been misidentified as *H. fijiensis* in the lowland region, giving a potential identification error rate of 1/208 (Dorey et al., 2020).

### Statistical analyses

We examined variation and co-variation in metabolic rate and frequency of gas exchange within *H. fijiensis* and among species across climatic space using linear mixed models using the nlme package (https://CRAN.R-project.org/package=nlme) in R Version 4.1.0 (http://www.R-project.org/). Using a strong inference approach, we used a suite of five models to test specific hypotheses that would support or reject the MCA and hygric hypotheses (Table 1; see rationale behind hypothesis tests in Box 1). The fifth model in each set was a null model that included altitude and no other environmental variables (Table 1). All models included log_10_-transformed dry body mass as a predictor variable as well as variables linked to the climate hypothesis of interest. Metabolic rate was also log_10_ transformed to ensure linear mixed effect model assumptions were met. Body mass and sex were correlated, so to avoid collinearity issues, we elected to remove sex as a predictor factor from our models as it is crucial to include body mass when examining variation in metabolic rate (Lighton, 2018). To deal with collinearity between the environmental variables in our models (and, as a result, high variance inflation factors, VIFs), especially between temperature, VPD and altitude, we modelled each environmental variable separately, except for a model that included temperature and precipitation of the driest month as VIFs were under 2 (Johnston et al., 2018). Models testing the hygric hypothesis included log_10_-transformed metabolic rate as a predictor variable to account for the effect of metabolic rate on frequency of gas exchange as they are positively correlated. Multispecies models testing the MCA and hygric hypotheses included species as a fixed factor within the models. Measurement block (sets of bees measured at the same time) and channel were included as random intercepts within each model.

We compared the Akaike information criterion (AIC) value of each model to determine which hypothesis best explained the variation in metabolic rate, frequency of gas exchange and how traits co-vary across abiotic variables as per Burnham and Anderson (2002) and White et al. (2007) using the MuMIn package (https://CRAN.R-project.org/package=MuMIn). We then evaluated the direction of the relationships between the traits of interest and abiotic variables within the best-fitting models to make inferences on whether metabolic rate and frequency of gas exchange are best explained by the MCA hypothesis and hygric hypothesis, respectively, and whether the relationship between metabolic rate and frequency of gas exchange remains consistent across abiotic variables or becomes uncoupled. All models included log_10_-transformed body mass as a predictor variable, and an interaction between log_10_-transformed body mass and species was included within the multispecies models. The multispecies models testing the hygric hypothesis also included an interaction between log_10_-transformed metabolic rate and species. Only species that had sample sizes over 10 individuals were included in the multispecies analysis (four species). The packages ggplot2 (Wickham, 2011) and cowplot (https://CRAN.R-project.org/package=cowplot) were used to produce data figures throughout the results.

We were unable to run a series of linear mixed effect models to examine whether the relationship (regression slope) between metabolic rate and frequency of gas exchange depends on the climatic conditions species inhabit. With small sample sizes and narrow elevational ranges for our two highland species (*n*=16 and *n*=23 for *H. groomi* and *H. tuiwawae*, respectively) we were underpowered to adequately test the three-way interaction between species, metabolic rate and climatic factor (each climatic factor was modelled in a separate model as per above) required to test how metabolic rate and frequency of gas exchange co-vary across climate space. However, we were able to estimate the slope of the relationship between frequency of gas exchange and metabolic rate for each species while accounting for body mass by running separate linear mixed effect models for each species. Frequency of gas exchange was the response variable, and log_10_-transformed metabolic rate and log_10_-transformed body mass were the fixed-factor predictor variables, and measurement block and channel were included as random intercepts. We then calculated the mean collection altitude, mean temperature of the coldest month, mean precipitation of the driest month and mean VPD for each species across the elevational gradient. We used linear models where the slope of the relationship between frequency of gas exchange and metabolic rate was the response variable, and each climatic variable was the fixed-factor predictor variable to evaluate whether the slope of the relationship between frequency of gas exchange and metabolic rate changes depends on aridity. The five models were compared using AIC as above.

Raw data are available in Dataset 1.

## RESULTS

### Testing the MCA hypothesis within and among species

**Fig. 3.**
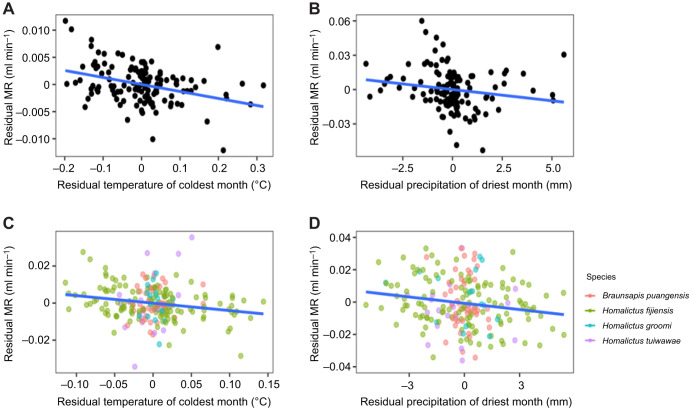
**Residual metabolic rate across residual temperature and precipitation.** (A,B) Residual metabolic rate (MR) of *H. fijiensis* (accounting for variation in body size) across residual average temperature of the coldest month (A) and residual precipitation of the driest month (B). (C,D) Residual metabolic rate of multiple Fijian bee species (accounting for variation in body mass) across residual temperature of the coldest month (C) and residual precipitation of the driest month (D). Note that residuals are presented for data visualisation purposes to highlight the effect of climatic variables on metabolic rate according to our models as per White et al. (2007); however, the raw data were analysed. Sample sizes: *H. fijiensis n*=125, *B. puangensis n*=44, *H. groomi n*=16, *H. tuiwawae n*=23.

The model that best explained variation in metabolic rate within *H. fijiensis* and across species included average temperature of the coldest month (*H. fijiensis*: estimate±s.e. −0.041±0.012, d.f.=92, *t*=−3.3, *P*<0.001; among species: estimate±s.e. −0.037±0.013, d.f.=167, *t*=−2.9, *P*=0.004), and average precipitation of the driest month as the test variables (*H. fijiensis*: estimate±s.e. −0.002±0.0005, d.f.=92, *t*=−3.6, *P*<0.005; among species: estimate±s.e. −0.002±0.0004, d.f.=167, *t*=−3.44, *P*<0.005) (Table 2 and see full model summaries in Tables S2 and S3). Metabolic rate was negatively correlated with both temperature and precipitation; therefore, an MCA-like pattern was observed (Figs 3A and 2B), but the MCA hypothesis was not supported in its strictest form as precipitation also explained a proportion of the variation in metabolic rate. Body mass was positively correlated with metabolic rate within *H. fijiensis* (estimate±s.e. 0.76±0.07, d.f.=92, *t*=11.6, *P*<0.001) and across bee species (estimate±s.e. 0.74±0.06, d.f.=167, *t*=12.37, *P*<0.001) (relationship between log_10_-transformed metabolic rate and log_10_-transformed body mass is shown in Fig. S3A–D).

**Table 2.** Testing the MCA hypothesis within *Homalictus fijiensis* and across Fijian bee species

Model environmental factors	d.f.	AIC	ΔAIC	*w_i_*	Marginal *R*^2^
*Homalictus fijiensis*	** **	** **	** **	** **	** **
***T*_min_+*P*_dry_**	**7**	**−84.53**	**0**	**0.962**	**0.55**
*P*_dry_	6	−75.99	8.54	0.015	0.51
VPD	6	−75.23	9.30	0.010	0.51
*T*_min_	6	−74.37	10.16	0.007	0.51
Altitude	6	−74.14	10.39	0.006	0.50
Multispecies	** **	** **	** **	** **	** **
***T*_min_+*P*_dry_**	**10**	**−96.17**	**0**	**0.92**	**0.48**
*P*_dry_	9	−89.65	6.52	0.04	0.46
VPD	9	−88.26	7.91	0.02	0.45
*T*_min_	9	−87.37	8.8	0.01	0.45
VPD	9	−86.99	9.18	0.01	0.45

Model comparison table for metabolic rate. The best-fitting model with lowest Akaike information criterion (AIC) value is in bold. All models also included log_10_-transformed body mass as a predictor variable. *w_i_* indicates AIC model weight.

### Testing the hygric hypothesis within and across species

**Fig. 4.**
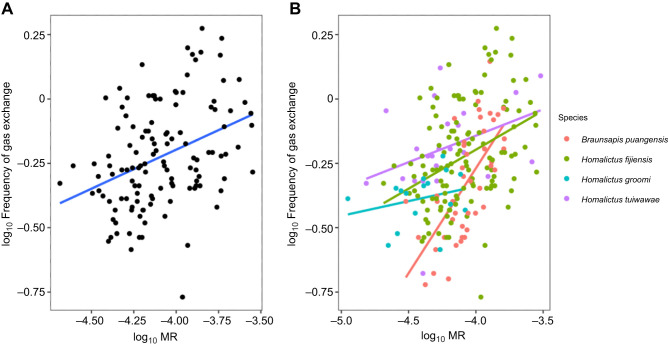
**Relationship between the frequency of gas exchange and metabolic rate within and among species.** Frequency of gas exchange (min^−1^) against metabolic rate (ml min^–1^) within *H. fijiensis* (A) and among species (B). Sample sizes: *H. fijiensis n*=125, *B. puangensis n*=44, *H. groomi n*=16, *H. tuiwawae n*=23.

The model that best explained variation in frequency of gas exchange within *H. fijiensis* and across species included only altitude as the explanatory environmental variable (Table 3). However, altitude did not have a significant effect on the frequency of gas exchange in *H. fijiensis* or among species (see Tables S4 and S5 for full model coefficient summaries). The best model was only marginally more supported than models that included precipitation of the driest month and temperature or precipitation of the driest month as the only environmental variable within the *H. fijiensis* models, and models that included precipitation of the driest month, temperature and VPD in the among species models, based on the ΔAIC values (<2 ΔAIC). However, none of the variables in the models that performed marginally worse than the best model were significantly correlated with frequency of gas exchange in either *H. fijiensis* or among species. Thus, the hygric hypothesis was not supported. Body mass was negatively correlated with frequency of gas exchange within *H. fijiensis* (estimate±s.e. −0.51±0.08, d.f.=92, *t*=−5.75, *P*<0.001) and among bee species (estimate±s.e. −0.43±0.07, d.f.=167, *t*=−5.96, *P*<0.001) (i.e. smaller bees have more rapid gas exchange frequencies than larger bees). Frequency of respiration was positively correlated with metabolic rate within *H. fijiensis* (estimate±s.e. 0.67±0.09, d.f.=92, *t*=7.74, *P*<0.001) and among species (estimate±s.e. 0.55±0.06, d.f.=167, *t*=9.05, *P*<0.001) (Fig. 4).

**Table 3.** Testing the hygric hypothesis within *Homalictus fijiensis* and across Fijian bee species

Model environmental factors	d.f.	AIC	ΔAIC	*w_i_*	Marginal *R*^2^
*Homalictus fijiensis*	** **	** **	** **	** **	** **
**Altitude**	**7**	**−84.99**	**0**	**0.37**	**0.34**
* P*_dry_+*T*_min_	8	−84.42	0.57	0.24	0.34
* P*_dry_	7	−83.26	1.73	0.16	0.33
VPD	7	−82.86	2.13	0.13	0.32
*T*_min_	7	−82.62	2.37	0.11	0.32
Multispecies					
** Altitude**	**10**	**−147.54**	**0.00**	**0.33**	**0.35**
* P*_dry_	10	−146.76	0.78	0.22	0.35
VPD	10	−146.30	1.24	0.18	0.35
* T*_min_	10	−146.26	1.28	0.17	0.35
*P*_dry_+*T*_min_	11	−145.58	1.96	0.11	0.35

Model comparison table for the frequency of gas exchange. The best-fitting model with the lowest AIC value is in bold. All models also included log_10_-transformed body mass, altitude and log_10_-transformed metabolic rate as fixed-factor predictor variables.

### Does the relationship between metabolic rate and frequency of gas exchange co-vary depending on the different climates species inhabit?

**Fig. 5.**
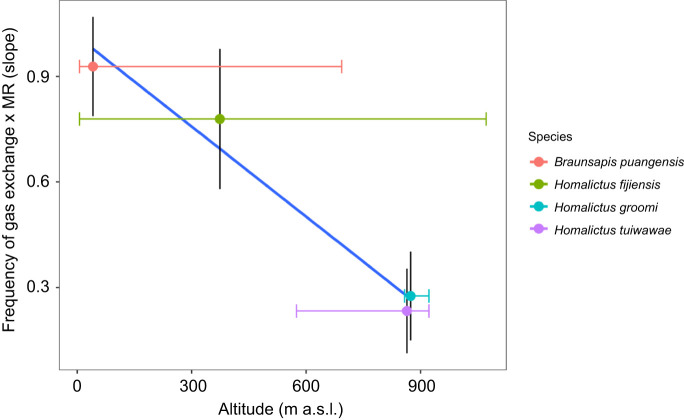
**Slope of the relationship (accounting for body mass) between frequency of gas exchange and metabolic rate depending on the mean elevation that species were collected from.** Circles are means, vertical error bars indicate the s.e.m. associated with each species slope, and horizontal error bars indicate the range of elevations that each species was collected across. *R*^2^=0.97. Sample sizes: *H. fijiensis n*=125, *B. puangensis n*=44, *H. groomi n*=16, *H. tuiwawae n*=23.

The model that included mean collection altitude best explained variation in the slope of the relationship between frequency of gas exchange and metabolic rate across species (*R*^2^=0.97) (Table 4; Table S6). The relationship between frequency of gas exchange and metabolic rate was steeper in species that occupy low elevation regions (*B. puangensis* and *H. fijiensis*) compared with species that are restricted to high elevations (*H. groomi* and *H. tuiwawae*) (Fig. 5).

**Table 4.** Trait covariation model comparison

Model environmental factors	d.f.	AIC	ΔAIC	*R* ^2^
**Altitude**	**3**	**−6.05**	**0.00**	**0.97**
VPD	3	−0.46	5.59	0.87
T_min_	3	−0.11	5.94	0.86
T_min_+P_dry_	4	0.98	7.03	0.89
P_dry_	3	7.49	13.54	0.08

Multispecies trait covariance model comparison (response variable is the slope of the relationship between frequency of gas exchange and metabolic rate). The best fitting model with lowest AIC value is in bold.

## DISCUSSION

Adaptive hypotheses that explain variation in physiological traits along climatic gradients make explicit predictions based on the expected direct effects of abiotic variables on physiological function. The MCA and hygric hypotheses predict that metabolic rates and gas exchange patterns evolve in response to the effects of temperature and water availability on these two traits, respectively (Addo-Bediako et al., 2002; White et al., 2007). Whether these hypotheses are supported within and among species and across environments has been the centre of debate over the last few decades (Addo-Bediako et al., 2002; Alton et al., 2024; Clarke, 2003; Messamah et al., 2017; Oladipupo et al., 2022; Rowe et al., 2022; White et al., 2007). However, despite metabolic rate and gas exchange patterns being inherently linked, how they covary across climatic conditions is rarely considered. Understanding how climate shapes traits and their relationships is likely to be critical for improving our understanding of the mechanisms that underpin species distributions and survival with further climate change (Hellmann and Pineda-Krch, 2007; Svensson et al., 2021). We discuss how metabolic rate and frequency of gas exchange vary across climatic gradients independently and then simultaneously to explore how these traits might evolve together.

We found that the MCA hypothesis was not supported in its strictest form in Fijian bees. Although we found a negative relationship between metabolic rate and temperature, as predicted by the MCA hypothesis, the best model that explained variation in metabolic rate also included precipitation. We expected that if precipitation did explain variation in metabolic rate, that metabolic rate would be lowest in dry environments and highest in wet environments as a mechanism to avoid desiccation (Box 1). However, we found that metabolic rate was negatively correlated with precipitation (a positive relationship with both temperature and precipitation is possible because of the elevational thermal/precipitation gradient and the longitudinal rain shadow on Viti Levu) (Figs 1 and 3B,D). Another recent study also found that environmental temperature and precipitation explain variation in metabolic rate (when tested at low temperatures, 18°C and 25°C, but not 30°C), where ants from colder and wetter climates had higher metabolic rates than those from warmer, drier environments (Willot et al., 2023). Unlike our study, however, Willot et al. (2023) found a positive relationship between metabolic rate and precipitation. Perhaps they detected a positive relationship between metabolic rate and precipitation because the ant species they examined exist across a broader climatic gradient than the Fijian bees in our study (ants were collected from two locations in Europe, but across the 13 species they tested, they differed greatly in their range limits, which is what the researchers used to extract climatic values). While we tested trait variation across a relatively narrow warm and humid climatic gradient, it remains important to determine whether macroevolutionary hypotheses hold even at a more micro-level. Finally, both temperature and precipitation have been found to influence species thermal limits (Riddell et al., 2023), so it is unsurprising that metabolic rate, which influences thermal limits (Padfield et al., 2016), would also be explained by both temperature and precipitation.

Variation in the frequency of gas exchange within *H. fijiensis* and across Fijian bee species was not significantly explained by any environmental variables included in our analysis (Table 3). Therefore, we do not find any empirical support for the hygric hypothesis within bees in Fiji. Perhaps the VPD in tropical Fiji is not desiccating enough (despite precipitation differences on either side of the rain shadow having a large effect on Fijian plant communities) for highly mobile nectarivorous bees to influence their rate of gas exchange. The occurrence of discontinuous gas exchange in these tropical pollinators therefore remains an open question. Discontinuous gas exchange has evolved independently at least five times (Marais et al., 2005), and thus there could be multiple mechanisms that explain why discontinuous gas exchange occurs in insects, and the variation in frequency of gas exchange we observe within species that breathe discontinuously (Chown, 2002; Matthews and White, 2011). The chthonic hypothesis (Lighton, 1998) posits that breathing discontinuously improves gas exchange in underground (high CO_2_) environments. Although this hypothesis, does not tend to garner much empirical support (Chown and Holter, 2000; White et al., 2007), including within a recent meta-analysis (Oladipupo et al., 2022), it could explain the evolution of discontinuous gas exchange in bees. *Homalictus* bee species nest in deep (often over 1 m deep) ground nests in large groups (Danforth and Ji, 2001), which could potentially create high CO_2_ environments. *Braunsapis puangensis* nest in stems above the ground, but many individuals will often live together within short linear nests (da Silva et al., 2016a,b), and they are known to plug their entrance holes with their abdomens to keep rain and predators out of the nest (C.R.B.d.S. and M.P.S., personal observation), which is a known behaviour in other allodapine bee species (Melna and Schwarz, 1994). Therefore, *B. puangensis* might also live in high CO_2_ nest environments, despite living above ground.

Alternatively, discontinuous gas exchange could be a conserved state across all bee species when they are in a state of rest (perhaps Fijian bees do not breathe discontinuously while they are in flight). Matthews and White (2011) proposed that discontinuous gas exchange occurs as a consequence of reduced or downregulated brain activity. *Homalictus* spp. and *B. puangensis* belong to different families, Halictidae and Apidae, respectively, and they both exhibited discontinuous gas exchange when placed in the dark metabolic rate chamber, as do other species within Apidae (Lighton and Lovegrove, 1990; Beekman and Van Stratum, 1999) and Megachilidae (Grula et al., 2021). Thus, perhaps variation in the frequency of gas exchange within and among species can be attributed to the degree of brain activity while at rest (Matthews and White, 2011), as well as metabolic rate and body mass (this study and Terblanche et al., 2008). Finally, the oxidative damage hypothesis (Bradley, 2000) suggests that discontinuous gas exchange evolves to reduce toxic effects of near-ambient intratracheal oxygen levels. Unfortunately, we have no way of evaluating whether this hypothesis could explain variation in the frequency of gas exchange in the current study and thus cannot speculate on whether our data might support it or not. However, the oxidative damage hypothesis seems to have very little empirical support from other studies (Matthews and White, 2011; White et al., 2007).

The relationship between frequency of gas exchange and metabolic rate changed across species in our study depending on their mean elevational range. The two species which dominate the lowland region, *B. puangensis* and *H. fijiensis*, had a much steeper relationship, where frequency of gas exchange increased more quickly with metabolic rate, than in the two highland species, *H. groomi* and *H. tuiwawae*. While the model that included altitude as the environmental factor had the lowest AIC value (see Table 4) and the highest *R*^2^ value (0.97), models that included VPD (*R*^2^=0.87), environmental temperature (*R*^2^=0.86), and environmental temperature and precipitation of the driest month (*R*^2^=0.89) also explained a large proportion of the variation in the slope of the relationship between frequency of gas exchange and metabolic rate. This suggests that temperature, aridity and another factor associated with elevation that we did not test could explain how frequency of gas exchange and metabolic rate co-vary across the environmental gradient. For example, perhaps elevation is correlated with nest depth or complexity, which could contribute towards nest CO_2_ levels if the chthonic hypothesis explains frequency of gas exchange.

While frequency of gas exchange and metabolic rate are linked (they are positively correlated across all species) we could be observing a physiological trait trade-off. Although we cannot determine whether the change in correlation between traits across the elevational gradient is due to correlational selection or a genetic correlation in this study, the fact that species from different environments have different trait relationships suggests that there is scope for metabolic rate and frequency of gas exchange to evolve independently of each other depending on environmental or other ecological factors. However, while the two traits might be evolving independently of each other, they remain correlated, and the extent to which each trait can evolve independently in response to variation in climate remains an exciting research question for the future.

We tested the MCA and hygric hypotheses, and how traits co-evolve across a tropical climatic gradient. While the MCA hypothesis aims to explain how organisms evolve to survive in cold environments, we found that even in a tropical environment across a narrow thermal range, there is evidence of metabolic thermal compensation. A potential limitation of our study is that these bees cannot be maintained in captivity long term and so were acclimated to common conditions for less than 24 h. We therefore cannot exclude effects associated with variation among collection sites. In addition, our experiment was only conducted on three species within the *Homalictus* genus (Halictidae family) and one species in the *Braunsapis* genus (Apidae family; therefore, the relationship between metabolic rate and frequency of gas exchange might differ across climatic gradients in other bee species, and indeed other kinds of insects. Finally, we tested how metabolic rate and frequency of gas exchange vary across an elevational gradient of ∼1000 m, but in these tropical mountains, variation in temperature and aridity is relatively small. However, it remains important to test whether macroevolutionary hypotheses hold across smaller spatial and taxonomic scales (i.e. within a single genera). Furthermore, testing macroevolutionary hypotheses in isolated environments, such as small islands, can help identify how specific variables influence species traits. For example, when macroevolutionary hypotheses are tested across broader latitudinal gradients, many factors other than climate also change, such as photoperiod and species interactions.

We believe it is critical to examine organisms as a mosaic of traits to improve our understanding of organismal responses to changing environments (Endler, 1995; Roff and Fairbairn, 2012; Svensson et al., 2021; Riddell et al., 2023). Understanding whether key adaptive hypotheses, such as the MCA hypothesis, are supported across species is crucial for predicting how climate change will impact species energetics in the future. However, variation in the strength of trait relationships (such as metabolic rate and frequency of gas exchange) could have implications for the ways in which species evolve with further anthropogenic climate change (Chantepie and Chevin, 2020). As organisms are composed of multiple interacting traits and adaptation is a multifactorial process, we believe it is important for future research to consider how multiple traits evolve across landscapes together as we move further into the Anthropocene.

## Supplementary Material

10.1242/jexbio.249948_sup1 Supplementary information

Dataset 1. Raw data
