# Supplementary material for: Temperature and precipitation explain variation in metabolic rate but not frequency of gas exchange in Fijian bees
Source: J Exp Biol. 2025 May 23;228(10):jeb249948. doi: 10.1242/jeb.249948 (PMC12148022; doi:10.1242/jeb.249948)
Supplement: Supplementary information [file jexbio-228-249948-s1.pdf]

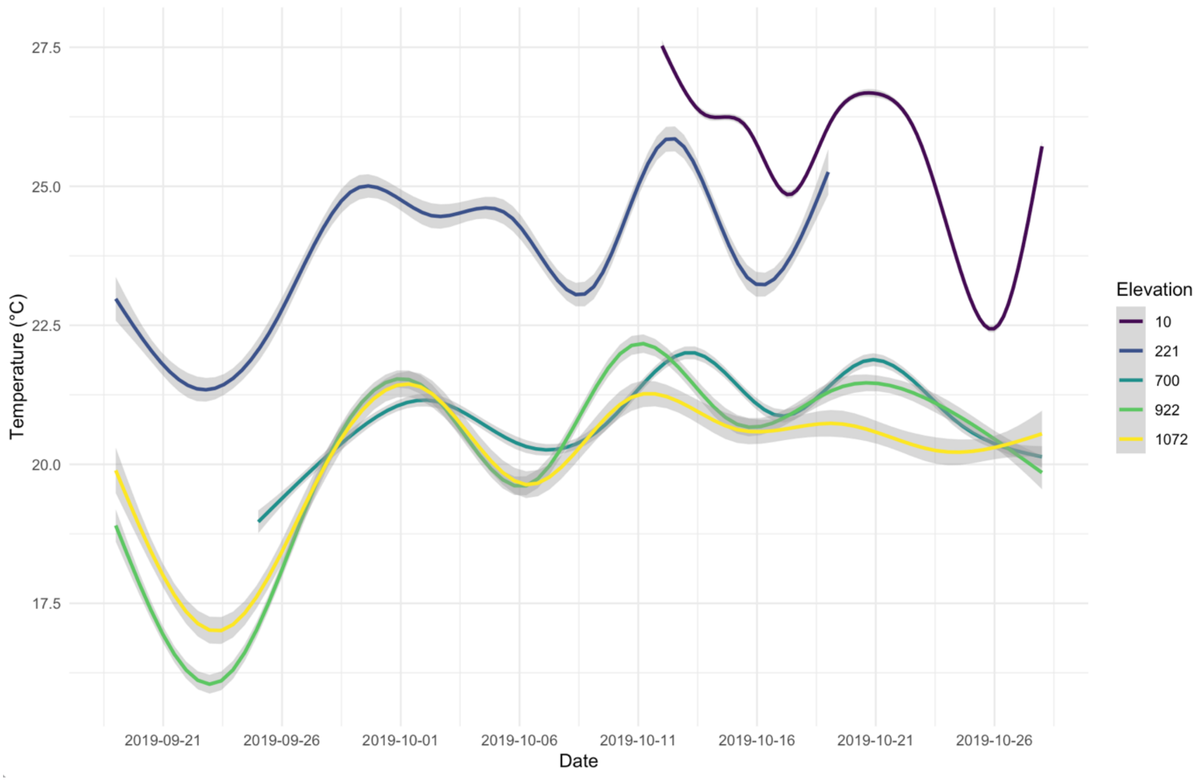

Fig. S1. Mean temperature collected from 5 sampling sites (at 10, 221, 700, 922 and 1072 m above sea level) across the elevational gradient with HOBO temperature loggers (temperature collected in the shade).

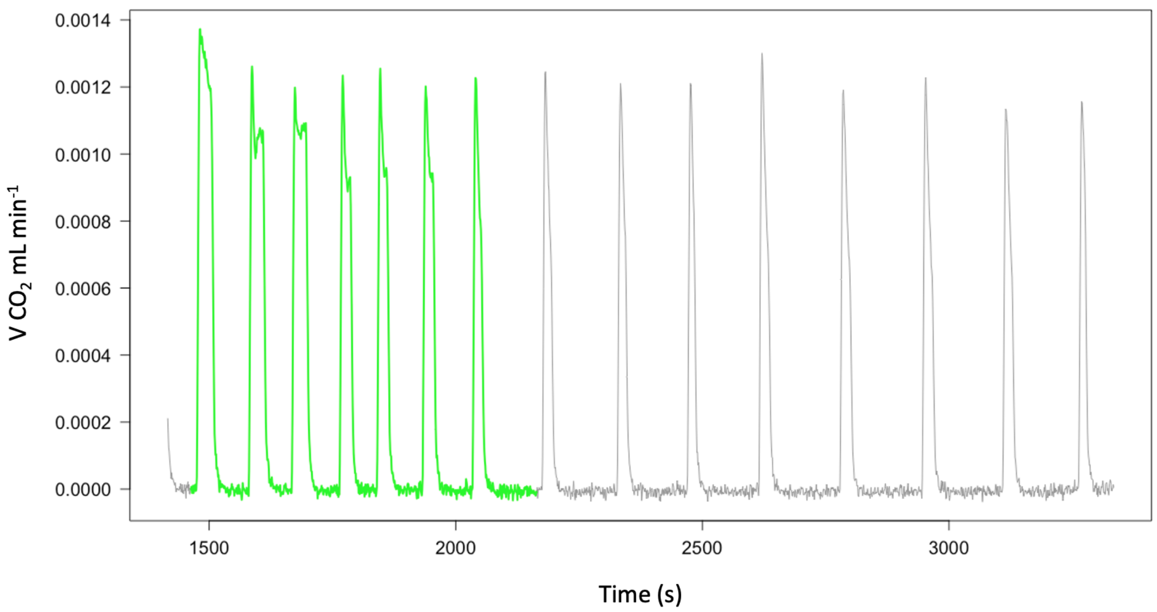

Fig. S2. An example recording of 7 full discontinuous gas exchange cycles (VCO_2, ml min-1) (highlighted in green) by an individual bee.

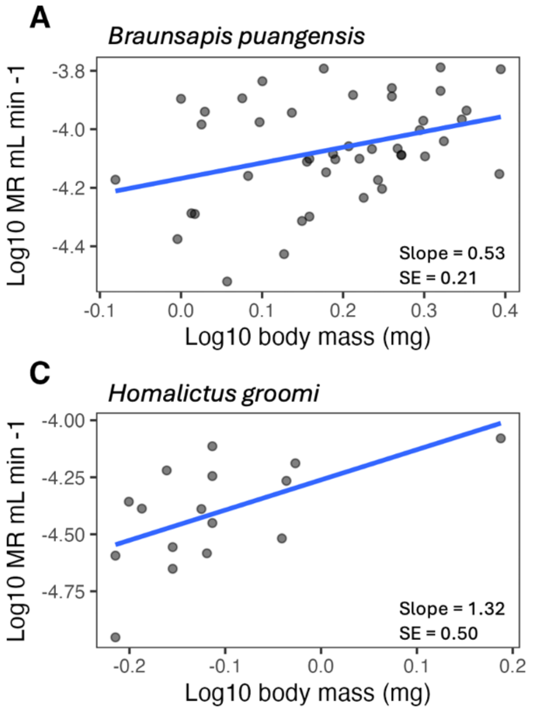

Fig. S3. Relationship between log10-transformed metabolic rate and log10-transformed body mass for *Braunsapis puangensis* (A), *H. fijiensis* (B), *H. groomi* (C) and *H. tuiwawae* (D). The slope and standard error (SE) of the relationship between metabolic rate and mass for each species is indicated for each species.

Journal of Experimental Biology • Supplementary information

Table S1. Summary of the abiotic variable ranges that each species was collected across.

Abiotic variables	*Braunsapis puangensis*	*Homalictus fijiensis*	*Homalictus groomi*	*Homalictus tuiwawae*
Minimum altitude (m a.s.l.)	6.0	6.0	858.0	575.0
Maximum altitude (m a.s.l.)	693.0	1072.0	922.0	922.0
Altitudinal range (m a.s.l.)	687.0	1066.0	64.0	347.0
Mean temp. July minimum (°C)	20.9	18.6	18.6	19.9
Mean temp. July maximum (°C)	22.9	23.1	19.9	20.4
July temp range (°C)	2.1	4.5	1.3	0.5
Mean precipitation minimum (mm)	87.1	57.6	103.0	103.0
Mean precipitation maximum (mm)	132.2	187.8	187.8	105.7
Precipitation range (mm)	45.1	130.1	84.8	2.8
Mean VPD July minimum (kPa)	0.32	0.10	0.10	0.22
Mean VPD July maximum (kPa)	0.56	0.56	0.22	0.27
VPD range (kPa)	0.24	0.46	0.12	0.05
Mean dry body mass (mg)	1.60	1.31	0.80	0.66
Sample size	44	125	16	23

Journal of Experimental Biology • Supplementary information

Table S2. Testing the MCA hypothesis within *Homalictus fijiensis*. Best fitting model summary (lowest AIC from strong inference model comparison) showing the effect of body mass, average temperature of the coldest month and average precipitation of the driest month on routine metabolic rate.

Coefficient	Estimate	SE	df	t-value	P-value
Intercept	-3.09	0.30	92	-10.59	< 0.001
Log_{10}(body mass)	0.76	0.07	92	11.57	< 0.001
**T_{min}**	**-0.041**	**0.04**	**92**	**-3.29**	0.0014
**P_{dry}**	**-0.0017**	**0.002**	**92**	**-3.63**	< 0.001

Table S3. Testing the MCA hypothesis across species. Best fitting model summary (lowest AIC from strong inference model comparison) showing the effect of body mass, species, an interaction between sex and species, average temperature of the coldest month and average precipitation of the driest month on routine metabolic rate.

Coefficient	Estimate	SE	df	t-value	P-value
Intercept	-3.16	0.33	167	-9.65	< 0.001
log10(mass)	0.76	0.07	167	11.23	< 0.001
Homalictus fijiensis	-0.007	0.04	167	-0.17	0.86
Homalictus groomi	-0.19	0.07	167	-2.63	0.009
Homalictus tuiwawae	-0.002	0.07	167	-0.03	0.978
**T_{min}**	**-0.037**	**0.013**	167	**-2.90**	**0.004**
**P_{dry}**	**-0.001**	**0.0004**	167	**-3.45**	**0.0007**

Table S4. Testing the hygric hypothesis within *Homalictus fijiensis*. Best fitting model summary (lowest AIC from strong inference model comparison) showing the effect of body mass, sex, altitude, and average precipitation of the driest month on ventilation rate.

Coefficient	Estimate	SE	df	t-value	P-value
Intercept	2.55	0.35	92	7.14	<0.001
Log_{10}(body mass)	-0.51	0.09	92	-5.76	<0.001
Log_{10}(MR)	0.67	0.09	92	7.74	<0.001
Altitude	-0.00008	0.00004	92	-1.72	0.089

Journal of Experimental Biology • Supplementary information

Table S5. Testing the hygric hypothesis among bee species. Best fitting model summary (lowest AIC from strong inference model comparison) showing the effect of body mass, sex, altitude, and average precipitation of the driest month on ventilation rate.

Coefficients	Estimate	SE	DF	t-value	P-value
Intercept	1.99	0.26	167	7.78	<0.001
log10(mass)	-0.43	0.07	167	-5.96	<0.001
Homalictus fijiensis	0.066	0.03	167	1.92	0.056
Homalictus groomi	-0.029	0.06	167	0.45	0.65
Homalictus tuiwawae	0.095	0.06	167	1.55	0.12
$Log_{10}MR$	0.55	0.06	167	9.05	<0.001
Altitude	-0.00005	0.00004	167	-1.16	0.248

Table S6. Assessing how frequency of gas exchange and metabolic rate co-vary across abiotic gradients across bee species. Best fitting model summary (lowest AIC from strong inference model comparison) coefficients.

Coefficients	Value	SE	df	t-value	P-value
Intercept	1.01	0.07	2	14.59	0.005
Altitude	-0.0008534	0.0001	2	-7.90	0.016

Dataset 1. Raw data

Available for download at
https://journals.biologists.com/jeb/article-lookup/doi/10.1242/jeb.249948#supplementary-data
